# New regulators of systemic iron homeostasis

**DOI:** 10.1038/s41392-021-00696-z

**Published:** 2021-07-21

**Authors:** Tomas Ganz

**Affiliations:** Center for Iron Disorders, Department of Medicine, David Geffen School of Medicine, UCLA, Los Angeles, CA USA

**Keywords:** Molecular medicine, Experimental models of disease

In a recent study published in *Blood*, Jiang et al.^1^ describe two new mechanisms that may contribute to systemic iron homeostasis. They first identify the methylcytosine dioxygenase Tet1 as an iron-dependent epigenetic regulator of multiple downstream target genes. Sorting through the target genes, they go on to show that one of them, *Rnf217*, is a long-sought E3 ubiquitin ligase that regulates the degradation of the unique cellular iron exporter ferroportin in response to its ligand, the master iron-regulatory hormone hepcidin.

Iron is an essential trace element whose organismal uptake, tissue distribution, and chemical reactivity are closely regulated to avoid the threat of iron deficiency at one extreme and toxicity from the excess of chemically-reactive forms of iron at the other extreme. Vertebrates have evolved a homeostatic system that controls body iron content by regulating intestinal iron absorption, and, once the iron enters the organism, strictly conserving it by internally recycling iron from senescent cells and minimizing iron losses from the organism. Occasional iron losses from the organism, usually caused by bleeding, are responded to by increased iron absorption to rebuild iron stores. When stores are adequate, iron absorption is inhibited. Genetic or acquired disorders of iron homeostasis range from iron deficiency, which causes anemia and depletion of iron-containing enzymes critical for many vital processes, to iron overload, wherein excess iron in tissues enhances the production of injurious reactive oxygen species that impair organ function and can cause death. Iron disorders affect a large proportion of the world’s population and contribute substantially to its burden of disease.

Cellular efflux of iron into plasma from absorptive enterocytes, iron-recycling macrophages, and iron-storing hepatocytes is under the control of the master iron-regulatory peptide hormone hepcidin, secreted by hepatocytes. Hepcidin binds to its receptor ferroportin, the unique cellular iron exporter highly expressed in the basolateral membranes of duodenal enterocytes, in iron-recycling macrophages, and in iron-storing hepatocytes, and controls ferroportin membrane concentration and ferroportin-mediated iron transfer to blood plasma.^2^ By these mechanisms, hepcidin controls intestinal iron absorption and body iron stores, as well as the concentration of iron in plasma and extracellular fluid. In this relatively constant interior environment, the distribution of iron to individual cells is governed by cell-autonomous iron uptake mechanisms.

**Fig. 1:**
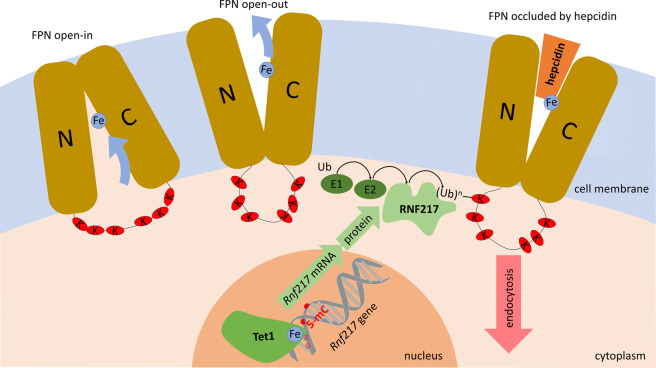
A model of ferroportin (FPN) regulation by Tet1 and RNF217. When hepcidin is not bound to it, FPN exports iron through the alternating access mechanism. Based on available structural information, FPN is depicted as two six-helix transmembrane bundles (N- and C-lobe) enclosing a central cavity and connected by a cytoplasmic lysine-rich loop. Hepcidin binding to FPN triggers a conformational change that makes lysines (K) accessible to ubiquitination by the E3 ubiquitin ligase RNF217. Ubiquitination of FPN targets it for endocytosis and proteolysis. Cellular concentrations of RNF217 are in part controlled by the iron occupancy of Tet1, an iron-dependent dioxygenase that activates the transcription of the *Rnf217* gene by demethylating its 5-methylcytosines (5-mC). In the presence of iron, intracellular concentrations of RNF217 rise and increase the endocytosis and proteolysis of FPN.

Ferroportin (SLC40a1) is a member of the solute carrier family of transporters. Ferroportin facilitates iron transport by a conformational flip-flop mechanism that alternates access to iron from the intracellular and extracellular faces of the transporter (Fig. 1). The two six-helix bundles of ferroportin enclose a central cavity through which the iron moves outward. What drives the outward directionality of iron transport is not yet understood but it is assumed that iron transport is coupled to the energetically more favorable transport of another ion or molecule. Hepcidin controls ferroportin activity through at least two mechanisms. First, it binds within the central cavity of ferroportin to occlude it when iron is transported from the cytoplasm to the extracellular space.^3^ This mode may require relatively high concentration of hepcidin to attain high occupancy of ferroportin. The second mode of regulation involves hepcidin-induced endocytosis and proteolysis of ferroportin. Here, hepcidin binding is thought to cause a conformational change in ferroportin, with consequent ubiquitination on a lysine-rich intracellular loop that connects the two halves of the ferroportin molecule.^4^

It is becoming clear that, besides the hormonal control by hepcidin-ferroportin ligand-receptor/transporter system, additional mechanisms contribute to systemic iron regulation, including hypoxia-inducible factor (HIF) prolyl hydroxylases that are sensitive not only to hypoxia but also to iron concentrations. These oxygen- and iron-sensing enzymes regulate HIF, and through it control the transcription of a number of genes involved in iron absorption, distribution, and utilization.^5^ Remarkably, Tet1 is also an iron- and 2-oxoglutarate-dependent dioxygenase and Jiang et al. present evidence that Tet1, through its ability to oxidize and demethylate 5-methylcytosines in DNA, functions as an iron-dependent epigenetic regulator of the E3 ubiquitin ligase RNF217 which in turn ubiquitinates ferroportin and thereby induces its endocytosis and proteolysis.

Ubiquitination is a regulatory mechanism by which a 76 amino acid protein ubiquitin is covalently attached to other proteins, usually to mark them for proteolytic degradation. It is carried out by a cascade of three enzymes (E1, E2, and E3 ubiquitin ligases) that, respectively, activate ubiquitin, transfer it, and ultimately ligate it to a lysine side chain in the target protein or subsequently to a lysine in the polyubiquitin chain on the target protein. Target protein specificity resides predominantly in the E3 ligase, as there are only 2 human genes for E1 and 35 for E2 but ~600–700 genes that encode E3 ligases. The authors present strong evidence that RNF217 contributes to ferroportin ubiquitination that drives hepcidin-induced ferroportin endocytosis and proteolysis. In the absence of RNF217 in vivo or in macrophages, hepcidin-induced ferroportin degradation was slower but not absent, suggesting that other E3 ligases may also regulate ferroportin degradation.

Importantly, the authors demonstrate that inactivation of either *Tet1* or *Rnf217* in mice produces small but clear-cut effects on systemic iron homeostasis, consistent with increased ferroportin activity. The phenotype is similar to that seen in mild forms of hereditary hemochromatosis caused by genetic regulatory lesions that impair iron-induced hepcidin synthesis. In the new model, Tet1 could function as an iron-sensing inducer of RNF217, enhancing RNF217-directed ferroportin degradation, which could increase the sensitivity of ferroportin to the effect of hepcidin (Fig. 1). The mechanism could help blunt duodenal iron absorption when tissue iron levels are already high. The emerging complexity of iron homeostasis may reflect the essential role of iron in many biochemical processes, and the evolutionary benefit of tight regulation of iron concentrations and tissue stores in a variety of biological contexts.
